# Ultrasonic Degradation Improves the In Vitro Utilization of Oolong Tea Pectic Polysaccharides: Structure Characterization and Multi-Omics Insights

**DOI:** 10.3390/foods15152749

**Published:** 2026-08-05

**Authors:** Meng Sun, Juqing Huang, Ruofei Zheng, Jiaxin Chen, Lingyue Zhong, Jie Li, Xuefang Guan, Qi Wang, Yafeng Zheng

**Affiliations:** 1 College of Food Science, Fujian Agriculture and Forestry University, Fuzhou 350002, China; yuta1010@163.com (M.S.); zhengruofei2001@163.com (R.Z.); cjx616479284@163.com (J.C.); 2 Institute of Food Science and Technology, Fuzhou 350003, China; jq_huang@zju.edu.cn (J.H.); lingyue_zhong@163.com (L.Z.); lijie282008@126.com (J.L.); guan-619@163.com (X.G.); 3 Key Laboratory of Processing of Subtropical Special Fruits and Vegetables and Mushrooms, Ministry of Agriculture and Rural Affairs, Fuzhou 350002, China; 4 Fujian Key Laboratory of Agricultural Product (Food) Processing, Fuzhou 350002, China

**Keywords:** tea polysaccharides, ultrasonic degradation, pectic polysaccharides, in vitro utilization, *Lactobacillus salivarius*, multi-omics, structure-function relationship

## Abstract

Pectic polysaccharides from tea residues represent a promising class of prebiotic dietary fibers. This study aimed to optimize the extraction of Oolong tea (*Camellia sinensis* ‘Foshou’) polysaccharides (FCTP), elucidate the structural alterations induced by ultrasonic degradation, and evaluate the consequent in vitro utilization using *Lactobacillus salivarius* BXP5. Enzyme-assisted extraction significantly improved the yield of FCTP to 11.10%, compared to 6.74% via conventional hot-water extraction. Ultrasonic treatment (120–240 W, 20–45 min) reduced the molecular weight (Mw) from 1079.9 kDa to 690.7–824.4 kDa, increased the uronic acid content, disrupted the triple-helix conformation, and transformed the polysaccharide into a looser and more water-accessible structure. Structural characterization by FTIR, methylation, and NMR further indicated that uFCTP remained an acidic pectic polysaccharide enriched in homogalacturonan (HG)-like domains. In vitro fermentation demonstrated that ultrasonically degraded FCTP (uFCTP) more effectively promoted BXP5 proliferation than native FCTP. Non-targeted metabolomics and proteomics revealed that uFCTP exerted more pronounced regulatory effects on nucleotide metabolism, carbon metabolism, and ribosomal biogenesis. Correlation analysis identified key metabolites (e.g., gallic acid, xanthosine monophosphate) tightly associated with differentially expressed proteins involved in carbohydrate utilization and cellular growth. These findings indicate that ultrasonic degradation is an effective physical modification strategy to improve utilization by *L. salivarius* BXP5 of tea-derived pectic polysaccharides by tailoring their molecular architecture for improved probiotic fermentation and metabolic cross-talk.

## 1. Introduction

Tea (*Camellia sinensis*) processing generates substantial quantities of tea residues that retain significant amounts of bioactive macromolecules, particularly polysaccharides [1]. Compared to small-molecule constituents such as polyphenols and caffeine, polysaccharides are less efficiently extracted during infusion and thus represent a high-value resource for valorization [2]. Among tea polysaccharides, pectic polysaccharides have attracted considerable attention due to their acidic heteropolysaccharide nature, characterized by a galacturonic acid (GalA)-rich backbone and diverse biological activities including immunomodulation, antioxidant capacity, and prebiotic functionality [3,4].

Foshou tea (Finger Citron tea), a distinctive oolong tea cultivar originating from Yongchun County, Fujian Province, China, is renowned for its unique aroma and traditional medicinal uses [5]. However, research on Foshou tea polysaccharides remains limited, and their structural features and prebiotic potential are poorly understood. Pectic polysaccharides from plant cell walls typically comprise homogalacturonan (HG), rhamnogalacturonan-I (RG-I), and rhamnogalacturonan-II (RG-II) domains, with HG being the most abundant structural motif [6,7]. The bioactivity of pectic polysaccharides is strongly governed by their molecular weight (Mw), degree of methylesterification, monosaccharide composition, and glycosidic linkage patterns [8,9].

Ultrasonic degradation has emerged as a green, efficient, and non-chemical physical modification technique to tailor polysaccharide structures. Acoustic cavitation generates transient high temperature, pressure, and shear forces that selectively cleave glycosidic bonds, reducing Mw and exposing active sites without altering the primary monosaccharide sequence [10,11]. Previous studies have demonstrated that ultrasonic treatment can enhance the antioxidant, anti-inflammatory, and prebiotic properties of various plant polysaccharides by improving solubility and increasing the relative abundance of uronic acid-rich fragments [12,13].

Prebiotics are defined as substrates that are selectively utilized by host microorganisms, conferring health benefits [14]. Polysaccharides serve as fermentable carbon sources for beneficial gut bacteria, promoting the production of short-chain fatty acids (SCFAs) and modulating microbial community structures [15,16]. The molecular weight and fine structure of polysaccharides critically influence their fermentation kinetics and microbial utilization efficiency [17]. Low-Mw pectins and oligosaccharides are generally more readily metabolized by lactic acid bacteria and bifidobacteria, leading to enhanced proliferation and metabolic activity [18,19].

Multi-omics approaches, integrating metabolomics and proteomics, provide a systems-level understanding of how prebiotic polysaccharides modulate probiotic physiology [20]. While the prebiotic effects of various plant polysaccharides have been reported, the molecular mechanisms underlying the interaction between ultrasonically degraded tea pectic polysaccharides and *Lactobacillus* species remain insufficiently characterized.

Therefore, the present study was designed to (i) optimize the extraction of FCTP using conventional hot-water and enzyme-assisted methods; (ii) systematically characterize the structural modifications induced by ultrasonic degradation using chemical analysis, chromatography, FTIR, SEM, methylation, and NMR spectroscopy; and (iii) evaluate the utilization of native and ultrasonically degraded FCTP on *Lactobacillus salivarius* BXP5 through in vitro fermentation coupled with non-targeted metabolomics and proteomics. This work provides a comprehensive structure–function framework for the development of Foshou tea polysaccharides as a novel potential microbiota-modulating polysaccharide ingredient.

## 2. Materials and Methods

**Figure 1:**
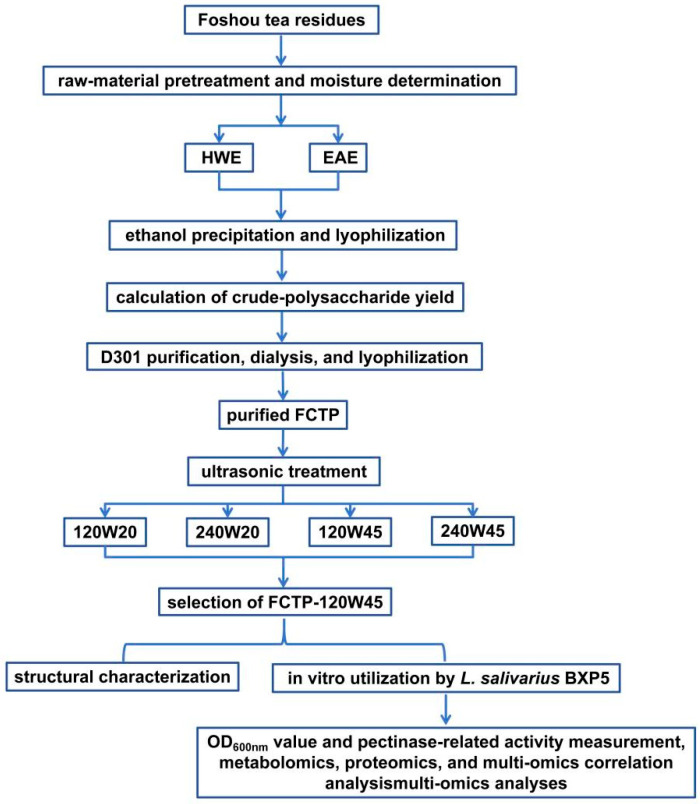
Schematic overview of this study design. Note: HWE and EAE denote hot-water extraction and enzyme-assisted water extraction, respectively; D301 denotes a macroporous anion-exchange resin.

The schematic overview of the study design is shown in Figure 1.

### 2.1. Materials and Reagents

Foshou tea residues were obtained from Yongchun County, Fujian Province, China. The moisture content of the initial tea material was determined by drying at 105 °C to constant weight and was found to be 5.8 ± 0.3%. Cellulase (100,000 U/g) and neutral protease (200,000 U/g) were purchased from Shandong Kotelon Enzyme Co., Ltd. (Linyi, China) and Jiangsu Ruiyang Bio-Technology Co., Ltd. (Wuxi, China), respectively. Monosaccharide standards, dextran standards, and other analytical-grade chemicals were obtained from Sigma-Aldrich (St. Louis, MO, USA) or Macklin Biochemical Co., Ltd. (Shanghai, China). *L. salivarius* BXP5 was isolated from human feces and identified through 16S rRNA gene sequencing. The strain was deposited in the China General Microbiological Culture Collection Center (CGMCC) under accession number 38198. Preliminary laboratory screening showed that the strain exhibited good tolerance to acidic conditions and bile salts, as well as antibacterial activity (BXP5 retained survival rates of 72.6% and 85.4% after exposure to pH 2.0 and pH 3.0 for 2 h, respectively, and 96.8% after treatment with 0.3% bile salts for 2 h; its cell-free supernatant produced inhibition zones of approximately 17–21 mm against representative pathogens). Furthermore, the potential pectinolytic activity of *L. salivarius* BXP5 was preliminarily demonstrated by its ability to grow in minimal medium containing citrus pectin as its sole carbon source. On the basis of these characteristics, *L. salivarius* BXP5 was selected for the present investigation.

### 2.2. Extraction and Optimization of FCTP

Two extraction protocols were compared: conventional hot-water extraction (HWE) and enzyme-assisted water extraction (EAE). For HWE, dried Foshou tea powder was extracted with distilled water at varying liquid-to-solid ratios (10–30 mL/g), extraction times (2–4.5 h), temperatures (65–85 °C), and particle sizes (60–140 mesh). For EAE, cellulase was added at concentrations ranging from 0.2% to 1.0% (*w*/*w*), with enzymatic hydrolysis conducted at 40–60 °C for 40–120 min prior to hot-water extraction. Single-factor experiments were followed by Box–Behnken design (BBD) with four factors at three levels to optimize extraction parameters. The extraction yield was calculated on a dry-weight basis as the mass of the lyophilized crude polysaccharides relative to the initial dry mass of the tea residue:

$$\mathrm{Extraction}\;\mathrm{yield}=\frac{W_{\mathrm{crude}\;\mathrm{polysaccharides}}}{W_{\mathrm{dry}\;\mathrm{residue}}}\times 100$$

where W_crude polysaccharides_ is the mass of the lyophilized crude polysaccharides and W_dry residue_ is the initial dry mass of the tea residue, as determined after moisture correction. All extraction yields were reported on a dry-weight basis.

Following extraction under optimized conditions, the crude extract was concentrated and precipitated with four volumes of 95% (*v*/*v*) ethanol. The precipitate was dissolved in distilled water (10 mg/mL), adjusted to pH 5.5, and loaded onto a pretreated D301 macroporous anion-exchange resin column (3.0 cm i.d. × 50 cm; bed height, 35 cm; bed volume (BV), 250 mL). The resin was sequentially treated with 1.0 mol/L HCl and 1.0 mol/L NaOH for 2.5 h each and rinsed to neutrality. Sample loading and deionized-water elution were performed at 2 BV/h, with 2 BV of eluate collected in 0.2-BV fractions. Carbohydrates, pigments, and salts were monitored at 490 nm, 280/420 nm, and by conductivity, respectively. Fractions with high carbohydrate and low pigment levels were pooled until the carbohydrate signal fell below 10% of the peak value. The pooled fractions were dialyzed against distilled water (MWCO 3500 Da) and lyophilized to obtain FCTP. The total sugar content of FCTP was determined using the phenol–sulfuric acid method [21].

### 2.3. Ultrasonic Degradation

The crude FCTP was dissolved in ultrapure water (10 mg/mL) and subjected to ultrasonic treatment using a probe-type ultrasonic cell disruptor (SCIENTZ-IID, Ningbo Scientz Biotechnology Co., Ltd., Ningbo, China) at different power/time combinations: 120 W/20 min, 120 W/45 min, 240 W/20 min, and 240 W/45 min. The resulting samples were dialyzed, lyophilized, and designated as FCTP-120W20, FCTP-120W45, FCTP-240W20, and FCTP-240W45, respectively. Untreated FCTP served as the control (FCTP-CK). The sample FCTP-120W45 was renamed uFCTP for subsequent studies. Among the four ultrasonically treated samples, FCTP-120W45 was selected for subsequent fermentation and multi-omics analyses because it exhibited the lowest apparent Mw (690.7 kDa) and the highest observed growth-promoting effect on BXP5 in preliminary screening (Supplementary Figure S4). The selection was based on these combined physicochemical and biological criteria and was not intended to imply that this condition or molecular weight is universally optimal.

### 2.4. Chemical and Physicochemical Analysis

Total sugar content was measured by the phenol–sulfuric acid method [21]. Protein content was determined using the Bradford assay [22]. The degree of methylesterification (DM) was assessed using a commercial pectin methylesterification kit (Shanghai Zeye Biotechnology Co., Ltd., Shanghai, China). Monosaccharide composition was analyzed using a Dionex ICS-5000+ ion chromatography system (Thermo Fisher Scientific, Sunnyvale, CA, USA) equipped with pulsed amperometric detection [23]. The weight-average molecular weight (Mw) and number-average molecular weight (Mn) were determined by high-performance gel permeation chromatography (HPGPC) using a Waters e2695 separation system equipped with a Waters 2414 refractive index detector (Waters Corporation, Milford, MA, USA), using dextran standards for calibration [24]. The residual polyphenol content of the purified polysaccharide fractions was determined using the Folin–Ciocalteu method with gallic acid as the standard [25]. The results showed that the purified FCTP contained 0.42 ± 0.05% (*w*/*w*) residual polyphenols, indicating substantial removal of co-extracted polyphenols during purification.

### 2.5. Spectroscopic and Microscopic Characterization

Fourier-transform infrared (FTIR) spectra were recorded on a Tensor 27 FTIR spectrometer (Bruker, Karlsruhe, Germany) using KBr pellets in the range of 4000–400 cm^−1^ [26]. Scanning electron microscopy (SEM) was performed after gold sputter coating to observe surface morphology using a Regulus 8230 scanning electron microscope (Hitachi High-Tech Corporation, Tokyo, Japan). The triple-helix conformation was investigated by the Congo red assay in NaOH solutions (0–0.5 mol/L), measuring the maximum absorption wavelength (λmax) from 400 to 700 nm [27].

### 2.6. Methylation and NMR Analyses

Methylation analysis was conducted according to Ciucanu and Kerek [28] with modifications. The permethylated polysaccharide was hydrolyzed, reduced, and acetylated to produce partially methylated alditol acetates (PMAAs), which were identified using an Agilent 7890B gas chromatograph coupled to an Agilent 5977A mass selective detector (Agilent Technologies, Santa Clara, CA, USA). For NMR spectroscopy, the sample was dissolved in D_2_O, and ^1^H NMR, ^13^C NMR, COSY, HSQC, HMBC, and NOESY spectra were acquired on a Bruker Avance spectrometer (Bruker, Karlsruhe, Germany) at 600 MHz [29].

### 2.7. In Vitro Fermentation

The effect of FCTP on the in vitro growth of *L. salivarius* BXP5 was evaluated in MRS medium containing different carbon sources. Activated *L. salivarius* BXP5 was inoculated into carbon-free MRS medium (CON, as blank control), MRS medium supplemented with 10 mg/mL glucose (GLC, as positive control), MRS medium supplemented with 10 mg/mL native FCTP (nFCTP), and MRS medium supplemented with 10 mg/mL ultrasonically degraded FCTP (uFCTP). The cultures were incubated at 37 °C for 0, 12, and 24 h. At each time point, an aliquot of bacterial suspension was collected to measure the optical density at 600 nm (OD_600_). The remaining culture was centrifuged, and the supernatant and bacterial pellet were separately collected and stored at −80 °C for subsequent pectinase activity, metabolomics, and proteomics analyses. Pectinase activity in the fermentation supernatants was determined using a commercial pectinase activity assay kit (Beijing Solarbio Science & Technology Co., Ltd., Beijing, China) according to the manufacturer’s instructions. Briefly, the substrate solution consisted of 1% (*w*/*v*) citrus pectin prepared in 0.1 M citrate buffer (citric acid-trisodium citrate, pH 3.5). The reaction mixture contained 100 μL of appropriately diluted fermentation supernatant and 400 μL of substrate solution. After incubation at 50 °C for 30 min, the reaction was terminated by adding 500 μL of 3,5-dinitrosalicylic acid reagent. The mixture was then heated in a boiling-water bath for 5 min and cooled to room temperature, after which the absorbance was measured at 540 nm. The amount of reducing sugar released was quantified using galacturonic acid as the standard. One unit (U) of pectinase activity was defined as the amount of enzyme required to release 1 μmol of reducing sugar, expressed as galacturonic acid equivalents, per minute under the assay conditions. Pectinase activity was expressed as U/mL of fermentation supernatant. All measurements were performed in triplicate.

### 2.8. Non-Targeted Metabolomics

Fermentation supernatants were collected at 24 h. Metabolites were extracted using ice-cold methanol/acetonitrile, and analyzed by UPLC–Q-Exactive Orbitrap MS (Thermo Fisher Scientific, Waltham, MA, USA) in both positive and negative ion modes [30]. Data were processed using Compound Discoverer software (version 3.3, Thermo Fisher Scientific, Waltham, MA, USA) for peak alignment, normalization, and annotation. Principal component analysis (PCA) and partial least squares discriminant analysis (PLS-DA) were performed. Differential metabolites were screened based on variable importance in projection (VIP > 1) and *p* < 0.05, and subjected to KEGG pathway enrichment analysis.

### 2.9. Proteomics Analysis

Proteomic analysis was conducted by Beijing Qinglian Bai’ao Biotechnology Co., Ltd. (Beijing, China). Briefly, bacterial pellets were lysed, and proteins were extracted and digested with trypsin. Peptides were analyzed by nano-LC–MS/MS using a DIA (data-independent acquisition) mode on a Q Exactive HF-X mass spectrometer (Thermo Fisher Scientific) [31]. Protein identification and quantification were performed using Spectronaut (version 18.6, Biognosys AG, Schlieren, Switzerland). Differentially expressed proteins (DEPs) were defined by fold change > 1.2 and *p* < 0.05. GO and KEGG enrichment analyses were conducted to elucidate biological functions.

### 2.10. Statistical Analysis

All experiments were performed in triplicate. Data were analyzed using GraphPad Prism 10.2.3 (GraphPad Software, San Diego, CA, USA) and expressed as mean ± standard deviation (SD). One-way ANOVA with Duncan test was used for multiple comparisons and *p* < 0.05 was considered statistically significant.

## 3. Results

### 3.1. Optimization of Extraction Conditions

The extraction yield of FCTP was significantly influenced by process parameters. For HWE, single-factor experiments indicated that increasing the liquid-to-solid ratio, temperature, and time initially enhanced the yield, but excessive conditions led to polysaccharide degradation (Supplementary Figure S1). BBD optimization predicted a maximum yield of 6.55% under the conditions of 240 min, 124 mesh, 80 °C, and 1:21 g/mL, with a validated yield of 6.74 ± 0.08% (Supplementary Table S1, Figure S2).

For EAE, cellulase pre-treatment effectively disrupted the cell wall matrix, facilitating the release of pectic polysaccharides. The optimal conditions predicted by BBD were 91 min, 0.8% enzyme dosage, 56 °C, and 1:25 g/mL, yielding a validated extraction rate of 11.10 ± 0.06% (Supplementary Table S2, Figure S3). The 4.36% increase in yield compared to HWE underscored the efficacy of enzymatic cell wall disruption for pectin recovery from tea residues.

### 3.2. Chemical Composition and Molecular Weight Distribution

The chemical composition and molecular weight of FCTP samples were summarized in Table 1. All FCTP fractions exhibited high total sugar content (90.56–99.96%) and low protein levels (<0.7%), confirming successful purification. Ultrasonic treatment significantly increased total uronic acid content (galacturonic acid + glucuronic acid) from 14.34% (FCTP-CK) to 23–24.6% in treated samples, indicating enrichment of acidic pectic domains. Degree of methylesterification (27.35–38.94%) classified all samples as low-methoxyl pectins.

Monosaccharide composition showed a clear structural shift after ultrasound treatment. In native FCTP, glucose (24.93%) was dominant, while uronic acid content was relatively lower (13.27%). After ultrasonication, glucose decreased (~12–15%), whereas galacturonic acid increased (~22–24%), indicating selective exposure/enrichment of HG-like regions.

Molecular weight analysis revealed a clear depolymerization trend: FCTP-CK (1079.9 kDa) > FCTP-120W20 (824.4 kDa) > FCTP-240W20 (757.0 kDa) > FCTP-240W45 (698.3 kDa) > FCTP-120W45 (690.7 kDa). The reduction in apparent Mw was consistent with partial depolymerization. However, the specific glycosidic linkages affected by ultrasound could not be determined from the current data.

### 3.3. Morphological Characterization

**Figure 2:**
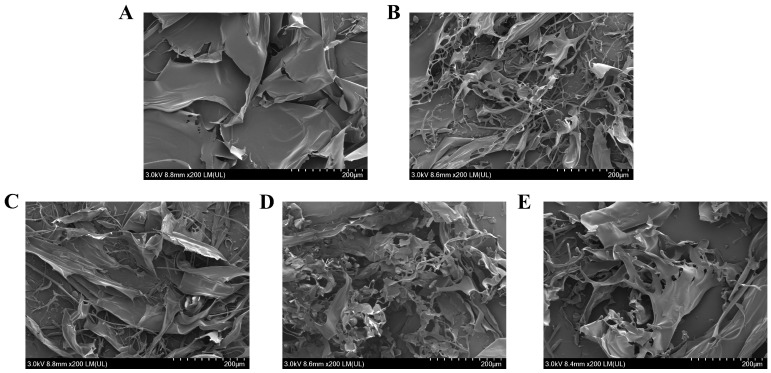
Scanning electron microscopy (SEM) images of FCTP samples. (**A**) FCTP-CK; (**B**) FCTP-120W20; (**C**) FCTP-240W20; (**D**) FCTP-120W45; (**E**) FCTP-240W45.

SEM analysis showed that native FCTP exhibited compact and smooth aggregated structures, while ultrasonically treated samples displayed increasingly porous, fragmented, and loose morphologies (Figure 2). These structural changes suggested that ultrasonic cavitation disrupts intermolecular aggregation and enhances surface accessibility, which may facilitate microbial enzymatic contact during fermentation.

### 3.4. FTIR and Congo Red Analysis

**Figure 3:**
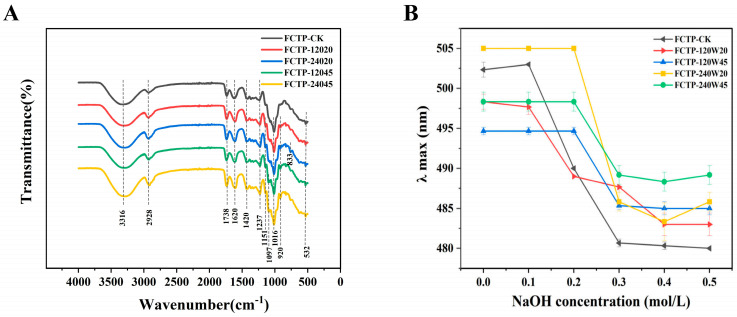
Spectroscopic analysis of FCTP. (**A**) FTIR spectra of FCTP-CK and ultrasonically degraded fractions. (**B**) Congo red assay showing the maximum absorption wavelength (λmax) as a function of NaOH concentration.

FTIR spectra (Figure 3A) confirmed the polysaccharide nature of all samples, with broad absorption bands at ~3316 cm^−1^ (O–H stretching) and ~2928 cm^−1^ (C–H stretching). Significant differences emerged in the fingerprint region. The untreated FCTP-CK showed weak absorption at ~1738 cm^−1^ (esterified C=O) and ~1620 cm^−1^ (COO^−^ asymmetric stretching). In contrast, uFCTP samples exhibited intensified peaks at 1738, 1620, and 1420 cm^−1^, alongside enhanced signals at 1237 and 1016 cm^−1^ corresponding to C–O stretching and pyranose ring vibrations. Enhanced absorption at 1738 cm^−1^ and 1620 cm^−1^ in ultrasonically treated samples indicated increased exposure of esterified and carboxyl groups, consistent with higher uronic acid content (Table 1).

Congo red analysis (Figure 3B) showed that native FCTP exhibited a red shift under alkaline conditions, suggesting partial ordered conformation. In contrast, ultrasonically treated samples lacked a consistent red shift, indicating disruption of higher-order triple-helix-like structures.

### 3.5. Structure Characterization of uFCTP Using Methylation and NMR Analyses

Methylation analysis identified 16 distinct glycosidic linkages in uFCTP (Supplementary Table S3). The predominant residue was 1,4-linked GalA (28.90 mol%), followed by 1,4-linked Gal (26.83 mol%), supporting the presence of a substantial HG-like domain. Terminal Araf (8.56 mol%), 1,5-linked Araf (3.52 mol%), and 3,6-linked Galp (5.27 mol%) indicated the presence of possible RG-I-associated arabinan and arabinogalactan motifs. Glucose-related linkages totaled 13.34 mol%.

**Figure 4:**
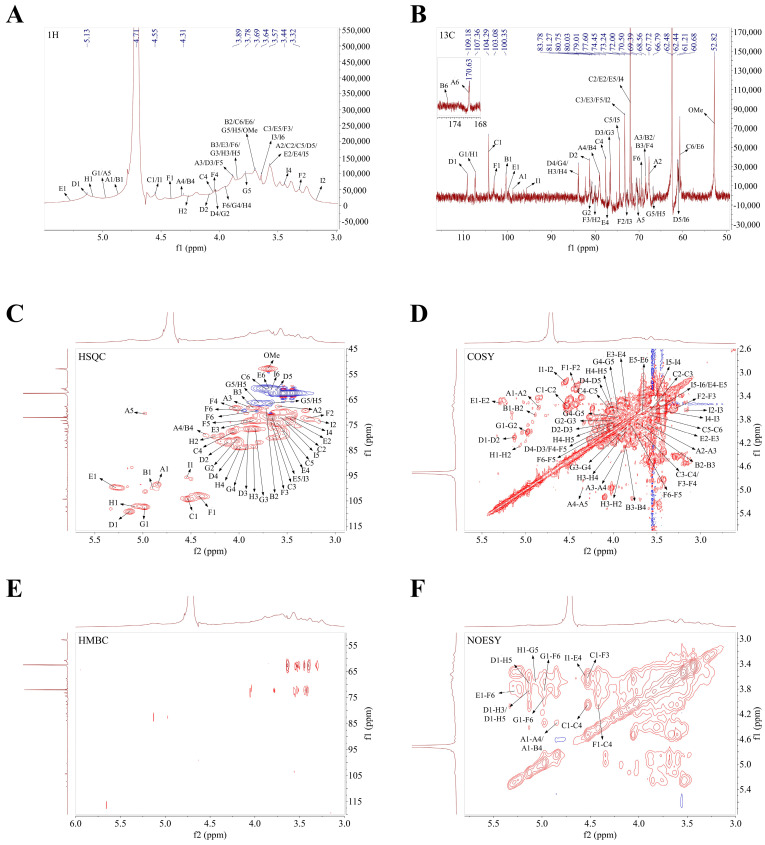
Nuclear magnetic resonance spectra of uFCTP: (**A**) ^1^H NMR; (**B**) ^13^C NMR; (**C**) HSQC; (**D**) COSY; (**E**) HMBC; (**F**) NOESY.

The ^1^H NMR spectrum of uFCTP (Figure 4A) showed that the proton signals were mainly distributed in the region of δ 3.0–5.5 ppm, with multiple anomeric signals appearing at δ 4.42, 4.53, 4.55, 4.85, 4.89, 4.98, 5.07, 5.13, and 5.29 ppm. The non-anomeric proton signals were mainly located in the range of δ 3.1–4.2 ppm. A strong signal near δ 4.71 ppm was assigned to the residual solvent peak, while the signal around δ 3.70 ppm was attributed to O-CH_3_ protons. In the anomeric carbon region, combined analysis of the ^13^C NMR and HSQC spectra (Figure 4B,C) identified the following cross-peaks: δ 4.85/98.53, 4.89/100.35, 4.53/104.29, 5.13/109.18, 5.29/99.73, 4.42/103.08, 4.98/107.36, 5.07/107.44, and 4.55/95.67 ppm, which were assigned to residues A–I, respectively. A signal near δ 52.82 ppm in the ^13^C spectrum was attributed to the carbon signal of the methoxyl group. Structural connectivity was further analyzed by combining ^1^H/^13^C assignments with COSY, HMBC and NOESY spectra (Figure 4D–F). No definitive long-range inter-residue correlations were observed in the HMBC spectrum. Therefore, the structural connectivity of uFCTP was mainly inferred from methylation data together with residue assignment and inter-residue NOESY correlations. According to the NOESY spectrum, cross-peaks were observed between A H1 and A H4, A H1 and B H4, C H1 and C H4, C H1 and F H3, D H1 and H H3/H5, E H1 and F H6, F H1 and C H4, G H1 and F H6, H H1 and G H5, and I H1 and E H4, supporting the presence of the proposed backbone and side-chain fragments. On the basis of methylation analysis, residue assignment, and literature comparison [32,33,34,35], residue A was assigned as →4)-α-D-GalpA-6-OMe-(1→, residue B as →4)-α-D-GalpA-(1→, residue C as →4)-β-D-Galp-(1→, residue D as α-L-Araf-(1→, residue E as →4)-α-D-Glcp-(1→, residue F as →3,6)-β-D-Galp-(1→, residue G as →5)-α-L-Araf-(1→, residue H as →3,5)-α-L-Araf-(1→, and residue I as β-D-Glcp-(1→. The detailed ^1^H and ^13^C chemical shift assignments for each residue were summarized in Table 2.

**Figure 5:**
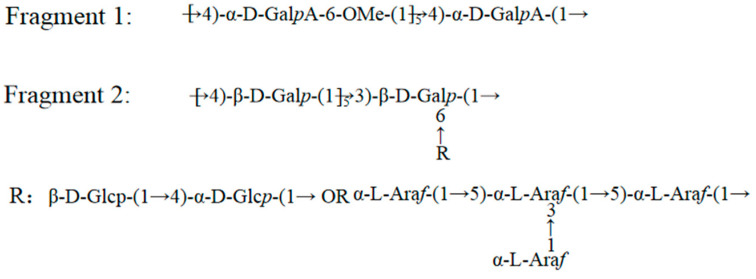
Tentative partial structural model of uFCTP.

Based on methylation analysis and 1D/2D NMR spectroscopy, uFCTP was tentatively characterized as a heterogeneous acidic pectin-like polysaccharide containing substantial HG-like GalA-rich regions and minor putative RG-I-associated, arabinan-like, and arabinogalactan-like motifs. The predominance of 4-linked GalpA residues indicated substantial homogalacturonan (HG)-like regions, whereas minor Rha-related linkages and branched Ara- and Gal-containing residues suggested the possible presence of RG-I-associated arabinan- and arabinogalactan-like motifs, including highly branched arabinan and β-(1→3,6)-linked galactan structures. However, the characteristic alternating GalA–Rha backbone of RG-I was not directly confirmed because Rha residues could not be unambiguously assigned in the NMR spectra and definitive long-range inter-residue correlations were lacking. Therefore, the precise sequences, domain organization, and covalent connections among these structural features remain unresolved, including whether they occur within a single polysaccharide molecule. Accordingly, a tentative partial structural model of uFCTP is proposed in Figure 5.

Taken together, the methylation and NMR data supported the presence of 4-linked GalA-rich segments, Gal-containing branched motifs, arabinan-related residues, and minor glucan-like components in uFCTP. However, because of substantial signal overlap and the absence of definitive long-range inter-residue HMBC correlations, the complete residue sequence and branching pattern could not be unambiguously established. Definitive assignment of the complete primary structure would require additional techniques such as partial acid hydrolysis, enzymatic digestion, and/or Smith degradation.

### 3.6. In Vitro Utilization of FCTP by L. salivarius BXP5

**Figure 6:**
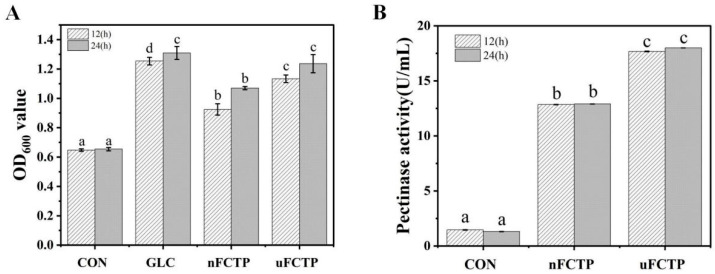
Growth performance (**A**) and pectinase activity (**B**) of *L. salivarius* BXP5 cultured with different carbon sources. CON: no carbon (blank control); GLC: glucose (positive control); nFCTP: native FCTP; uFCTP: ultrasonically degraded FCTP. Data are presented as mean ± SD (*n* = 3). Different letters indicate significant differences (*p* < 0.05).

The ability of FCTP to support the growth of *L. salivarius* BXP5 was assessed by monitoring bacterial growth in media containing different carbon sources (Figure 6A). The OD_600_ results show that uFCTP promoted significantly higher growth of *L. salivarius* BXP5 compared with native FCTP at 12 h (*p* < 0.05). At 24 h, the OD value of uFCTP group was also significantly higher than that of nFCTP group (*p* < 0.05), indicating enhanced fermentability after ultrasonic treatment. The results demonstrated that ultrasonically treated FCTP exhibited a superior in vitro growth-promoting effect on *L. salivarius* BXP5.

The effects of differently treated FCTP samples on the pectinase activity of *L. salivarius* BXP5 are shown in Figure 6B. After 12 h of fermentation, both the nFCTP and uFCTP groups exhibited significantly higher pectinase activity than the CON group (*p* < 0.05), and the pectinase activity in the uFCTP group was significantly higher than that in the nFCTP group (*p* < 0.05). After 24 h, pectinase activity further increased in both groups compared with that at 12 h. Specifically, the pectinase activity reached 12.91 ± 0.01 U/mL in the nFCTP group and 18.00 ± 0.01 U/mL in the uFCTP group. Overall, the pectinase activity in the uFCTP group remained consistently higher than that in the nFCTP group throughout fermentation, suggesting that ultrasonic treatment reduced the molecular weight and improved the enzymatic accessibility of FCTP, thereby facilitating its utilization by *L. salivarius* BXP5.

### 3.7. Metabolomic Responses

**Figure 7:**
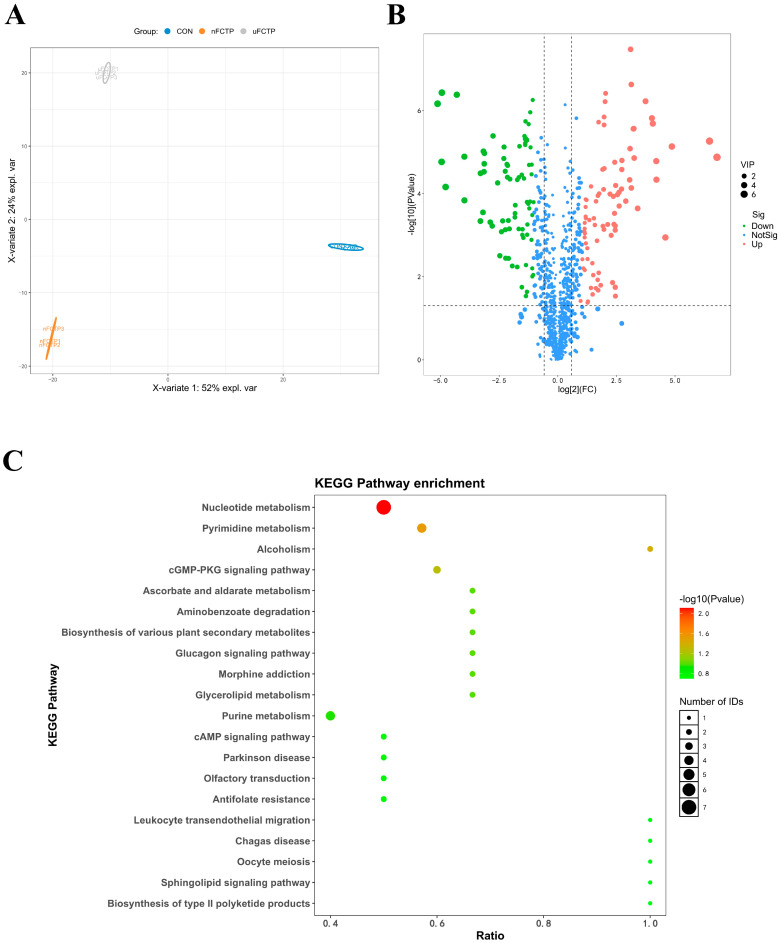
Metabolomic analysis of BXP5 fermentation products. (**A**) PLS-DA score plot of CON, nFCTP, and uFCTP groups; (**B**) Volcano plot of differential metabolites for uFCTP vs. nFCTP group; (**C**) KEGG pathway enrichment analysis of differential metabolites for uFCTP group vs. nFCTP group. nFCTP: native FCTP; uFCTP: ultrasonically degraded FCTP.

Partial least squares discriminant analysis (PLS-DA) revealed a clear separation among the control (CON), nFCTP, and uFCTP groups in the supervised discriminant model (Figure 7A), indicating that the different treatments had a significant effect on metabolic features. The volcano plot of differential metabolites for uFCTP vs. nFCTP group (Figure 7B) showed that compared to nFCTP, uFCTP treatment resulted in 165 differential metabolites (86 upregulated, 79 downregulated).

KEGG pathway enrichment (Figure 7C) highlighted that uFCTP versus nFCTP comparison revealed significant perturbations in nucleotide metabolism, pyrimidine metabolism, and purine metabolism. Among the differential metabolites, several key compounds exhibited significant changes in the uFCTP group, including gallic acid (VIP > 1, *p* < 0.05), xanthosine monophosphate (a purine nucleotide precursor), adenine, cyclic AMP, and lithocholate 3-O-glucuronide (a bile acid derivative), suggesting coordinated alterations in nucleotide metabolism, energy signaling, and lipid metabolism.

### 3.8. Proteomic Responses

**Figure 8:**
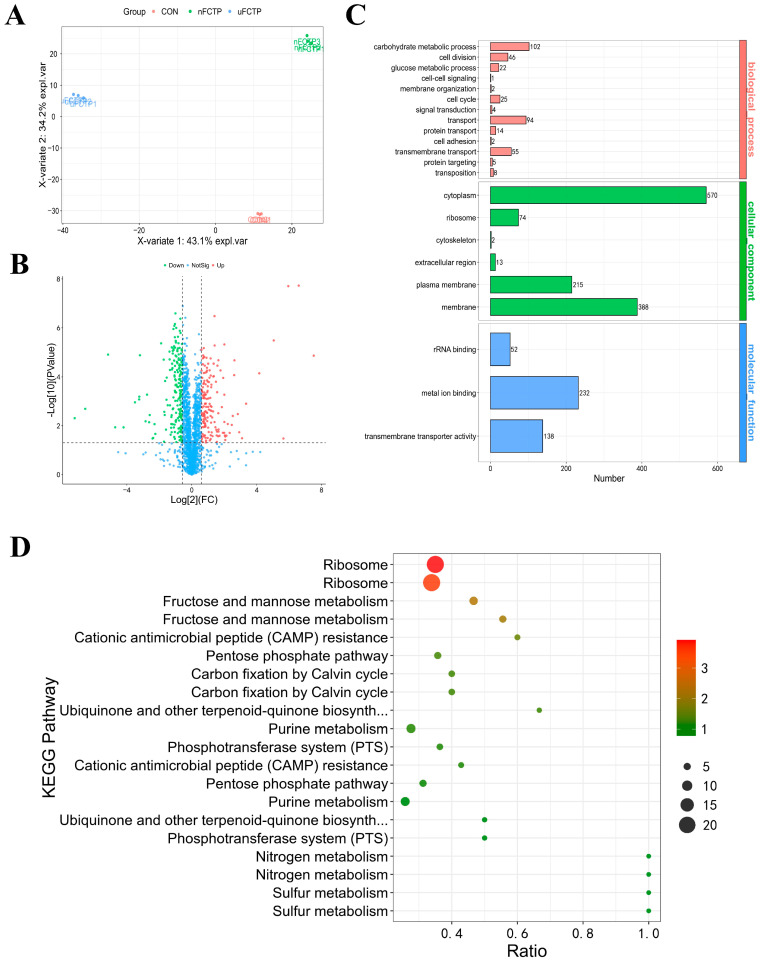
Proteomic analysis of *L. salivarius* BXP5 under different treatments. (**A**) PLS-DA score plot of CON, nFCTP, and uFCTP groups; (**B**) Volcano plot of differential proteins for uFCTP vs. nFCTP group; (**C**) GO enrichment analysis of differential proteins for uFCTP group vs. nFCTP group; (**D**) KEGG pathway enrichment analysis of differential proteins for uFCTP group vs. nFCTP group. nFCTP: native FCTP; uFCTP: ultrasonically degraded FCTP.

Proteomic profiling corroborated the metabolomic findings. The PLS-DA results in Figure 8A showed that X-variates 1 and X-variates 2 explained 43.1% and 34.2% of the variance, respectively. The three sample groups displayed clear separation in the two-dimensional space, with high within-group clustering and large between-group distances, further indicating stable discrimination among the groups. In particular, the uFCTP group was distinctly separated from both the CON group and the nFCTP group along X-variate 1, and it also showed evident separation from the nFCTP group along X-variate 2. Overall, uFCTP treatment induced a more pronounced and uniquely directional shift in the overall sample structure, suggesting that it exerted a stronger regulatory effect and more distinct intergroup discriminative characteristics than nFCTP treatment. Differential protein analysis identified 359 DEPs (160 up, 199 down) for uFCTP vs. nFCTP (Figure 8B).

GO enrichment analysis (Figure 8C) indicated that DEPs were predominantly involved in carbohydrate metabolic processes, cell division, translation, signal transduction, and transmembrane transport. Molecular functions were enriched in metal ion binding, rRNA binding, and transmembrane transporter activity. KEGG analysis (Figure 8D) revealed that compared to nFCTP, uFCTP significantly enriched pathways related to ribosome, thiamine metabolism, fructose and mannose metabolism, and the pentose phosphate pathway.

### 3.9. Integrated Multi-Omics Analysis

**Figure 9:**
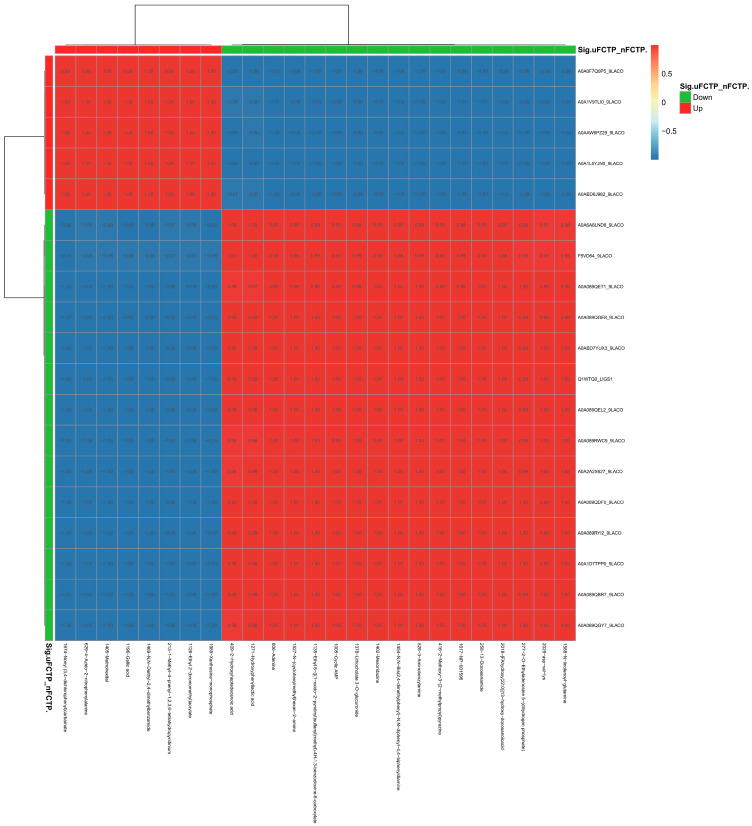
Top 30 positive correlations between differential metabolites and proteins in the uFCTP versus nFCTP comparison. nFCTP: native FCTP; uFCTP: ultrasonically degraded FCTP.

Correlation analysis between the top 30 differential metabolites and proteins revealed strong associations in uFCTP vs. nFCTP comparison groups (Figure 9). Key metabolites including gallic acid, xanthosine monophosphate, adenine, cyclic AMP, and lithocholate 3-O-glucuronide were tightly correlated with proteins involved in carbohydrate-active enzymes (CAZymes), ABC transporters, and ribosomal subunits.

## 4. Discussion

The substantial improvement in FCTP yield from 6.74% (HWE) to 11.10% (EAE) underscores a fundamental limitation of conventional thermal extraction for pectic polysaccharides from lignocellulosic tea matrices. The tea cell wall is a complex composite of cellulose microfibrils embedded in a pectin-rich matrix cross-linked with hemicellulose and structural proteins [36,37]. Hot-water extraction mainly relies on diffusion-driven mass transfer, which is insufficient to disrupt these structural barriers. In contrast, cellulase hydrolyzes β-1,4-glycosidic bonds in cellulose, effectively loosening the structural scaffold and facilitating the release of pectic domains. This enzymatic disruption mechanism aligns with established models of plant cell wall deconstruction, in which cellulose accessibility is the key limiting factor [38]. The 64.7% relative increase in yield achieved herein positions EAE as a scalable and economically viable strategy for industrial valorization of tea residues, particularly given the mild operating conditions (56 °C, 91 min) that minimize thermal degradation of sensitive pectic structures. It should be noted that tea is naturally rich in polyphenols, which are well documented to modulate gut microbiota composition and activity. Although the purification procedure (D301 macroporous resin treatment, dialysis, and lyophilization) effectively reduced the residual polyphenol content to below 0.5% (*w*/*w*), the potential minor contribution of co-extracted phenolic compounds to the observed effects on bacterial growth cannot be completely excluded. Future studies should include a polyphenol-depleted control or employ specific polyphenol scavengers to definitively isolate the polysaccharide-specific effects.

Ultrasonic treatment induced systematic physicochemical modifications of FCTP, including reduced molecular weight, increased uronic acid content, and altered supramolecular organization. The decrease in Mw from ~1080 kDa to 690–824 kDa suggests partial cleavage of glycosidic linkages under cavitation-induced shear forces. The concurrent enrichment of GalA indicated a relative preservation and exposure of GalA-rich domains, which are more rigid and structurally ordered compared to RG-I region [39,40]. However, direct evidence for selective side-chain cleavage requires comparative methylation analysis of native and degraded fractions, which was not performed in this study. The increase in uronic acid content enhances the physicochemical and biological functionality of uFCTP. GalA residues introduce carboxyl groups that increase negative charge density, which is important for bacterial adhesion and enzyme recognition. Many *Lactobacillus* species possess pectinolytic enzyme systems capable of targeting HG backbones [41]. Therefore, the enrichment of GalA-rich pectic segments in uFCTP likely improves substrate compatibility for *L. salivarius* BXP5. This structural bias supports the concept that polysaccharide bioactivity is governed not only by composition but also by domain organization, where “substrate architecture” determines microbial accessibility. Congo red analysis indicated that native FCTP possesses ordered supramolecular structures, while ultrasonic treatment may disrupt this organization. The loss of triple-helix-related spectral shifts suggests the breakdown of interchain hydrogen bonding networks typical of rigid polysaccharide assemblies [42]. Such structural relaxation may enhance solubility and enzyme accessibility. Although ordered conformations can contribute to stability, they often hinder enzymatic hydrolysis due to steric shielding [43]. The transition toward a more flexible and disordered conformation therefore likely facilitates microbial degradation [44]. Consistently, SEM images further confirmed increased porosity and fragmentation, which expands the surface area available for microbial interaction. Experimentally, ultrasound treatment was associated with a reduction in apparent molecular weight, changes in relative monosaccharide composition, altered FTIR and Congo red responses, and a more fragmented surface morphology. These observations support partial depolymerization and conformational rearrangement. The frequently proposed effects of acoustic cavitation, including local shear-induced chain scission and disruption of intermolecular aggregation, provide plausible explanations for these changes. However, the present experiments did not directly identify the cleaved glycosidic bonds or quantify the delivered acoustic energy. Therefore, these mechanisms should be regarded as interpretations rather than direct experimental observations.

Methylation and linkage analysis revealed that uFCTP is a GalA-rich pectic segment dominated by HG-like regions with possible RG-I-associated motifs. The presence of both methylesterified and non-methylesterified GalA residues suggests heterogeneous esterification patterns, which may provide multiple enzymatic cleavage sites for pectin-modifying enzymes [45]. In addition, arabinan and galactan side chains contribute to structural heterogeneity, which increases the diversity of fermentable substrates available to gut microbiota [46]. The coexistence of α- and β-linked residues further indicated biosynthetic complexity and may contribute to gradual fermentation behavior due to multi-enzyme requirements.

In vitro fermentation results confirmed that uFCTP significantly enhances the growth of *L. salivarius* BXP5 compared with native FCTP. This improvement is closely associated with increased substrate accessibility and improved enzymatic degradation efficiency. Higher pectinase activity in the uFCTP group indicated more active microbial utilization of polysaccharide substrates [47]. Compared with nFCTP, the lower molecular weight and higher GalA content of uFCTP may reduce diffusion barriers and enhance electrostatic interactions with bacterial surfaces, facilitating substrate uptake [48]. These findings are consistent with the “Goldilocks principle” of polysaccharide fermentability, where intermediate molecular sizes balance accessibility and sustained metabolic activity [49].

Multi-omics analysis further suggested that uFCTP might induce a stronger metabolic activation profile than nFCTP. The enrichment of nucleotide metabolism, particularly pyrimidine and purine metabolism, indicated enhanced biosynthetic demand associated with rapid bacterial proliferation. The elevated nucleotide levels indirectly suggest potential enhancement of the pentose phosphate pathway (PPP), which supplies ribose-5-phosphate for nucleotide synthesis. PPP activation also provides NADPH for reductive biosynthesis, supporting anabolic growth states. These changes suggest that uFCTP may act not only as a carbon source but also as a metabolic trigger that promotes cellular growth programs.

The elevated levels of amino sugars and other glycan-related metabolites in uFCTP fermentations suggest enhanced cell wall biosynthesis during active growth [50]. The presence of 2′-fucosyllactose, a human milk oligosaccharide analog, is intriguing; while not directly utilized by BXP5, it may function as a metabolic signaling molecule or competitive inhibitor of fucosidases, indirectly modulating glycan metabolism. Proteomic analysis further confirmed increased ribosomal biogenesis, indicating elevated translational activity (uFCTP vs. nFCTP). In addition, upregulation of thiamine metabolism suggests enhanced energy metabolism and cofactor availability, supporting sustained microbial growth [51]. Furthermore, the enrichment of cell division-related proteins aligns with the observed enhanced proliferation of BXP5 in the uFCTP group (Figure 6A), indicating that uFCTP not only provides carbon sources but also activates cellular replication programs. The involvement of signal transduction proteins suggests that uFCTP may trigger regulatory cascades that coordinate carbohydrate sensing, metabolic flux allocation, and growth responses, although the specific signaling mechanisms in *L. salivarius* require further investigation.

Integration of metabolomic and proteomic data reveals a coordinated response to uFCTP. Carbohydrate uptake via transporters is coupled with glycolysis and PPP flux, while downstream pathways support nucleotide, lipid, and protein biosynthesis. Compared with nFCTP, uFCTP induces a more coherent metabolic network (Supplementary Figures S5 and S6), indicating higher substrate efficiency and metabolic synchronization.

Overall, ultrasonic degradation may improve the utilization of FCTP by *L. salivarius* BXP5, potentially through structural loosening, increased GalA exposure, enhanced enzymatic accessibility, and changes in metabolic pathways associated with bacterial growth. This integrated structure–function–metabolism relationship may provide a mechanistic basis for the development of ultrasound-modified pectic polysaccharides as potential prebiotic substrates.

These findings suggest that uFCTP-BXP5 combinations may have potential as synbiotic formulations, although this requires validation in animal models and clinical trials. The demonstration that ultrasonic degradation enhances both the yield and growth-promoting effect on BXP5 of FCTP establishes a scalable manufacturing pipeline from tea waste to functional ingredient. The optimal ultrasonic conditions identified in this study (120 W for 45 min) may provide a basis for future process optimization in industrial flow-through or batch sonication systems.

Finally, the present study highlights the industrial potential of combining enzymatic extraction and ultrasonic modification to convert tea processing residues into high-value polysaccharide ingredients with potential prebiotic properties. The optimized ultrasonic conditions identified herein provide a scalable processing window suitable for industrial adaptation. However, although the purification procedure effectively removed the majority of polyphenols, the potential minor contribution of co-extracted phenolic compounds to the observed growth-promoting effects on BXP5 cannot be completely excluded. Future studies should include a polyphenol-depleted control or use specific polyphenol scavengers to definitively isolate the polysaccharide-specific effects. The present findings were obtained using a single probiotic strain under in vitro conditions, and therefore cannot directly represent the effects of uFCTP on complex gut microbiota communities or human health. Further validation in complex gut microbiota systems and in vivo models is required to confirm ecological relevance and translational efficacy. Future research should also explore the dose–response relationship between uFCTP and BXP5, the stability of uFCTP under gastrointestinal conditions using simulated digestion models, and the anti-inflammatory efficacy of the uFCTP-BXP5 potential synbiotic combination in colitis animal models. The latter is particularly promising given the established role of pectic oligosaccharides in modulating intestinal immune homeostasis and the demonstrated anti-inflammatory potential of *L. salivarius* strains [52,53].

## 5. Conclusions

This study presents a comprehensive investigation into the extraction, structural modification, and growth-promoting effect on BXP5 of Foshou tea pectic polysaccharides. Enzyme-assisted extraction provided a superior yield (11.10%) compared to conventional hot-water extraction. Ultrasonic degradation effectively tailored the physicochemical properties of FCTP by reducing molecular weight, increasing uronic acid content, disrupting the triple-helix conformation, and enriching the HG-like backbone. These structural modifications translated into significantly enhanced strain-specific utilization, as evidenced by the robust proliferation of *L. salivarius* BXP5. Multi-omics analysis revealed that uFCTP profoundly reshaped the metabolome and proteome of BXP5, particularly upregulating nucleotide metabolism, pentose phosphate pathway, and ribosomal biogenesis. The integrated metabolite–protein network highlights a coordinated cellular response that enhances carbohydrate utilization and energy production. These findings suggest that ultrasonic degradation may serve as a green approach for tailoring tea polysaccharides to improve their strain-specific utilization, highlighting their potential use in microbial culture formulations and functional foods designed to support gut microbial metabolism.

## Figures and Tables

**Table 1:** Chemical composition and molecular weight distribution of FCTP samples.

Sample	FCTP-CK	FCTP-120W20	FCTP-240W20	FCTP-120W45	FCTP-240W45
Total Sugar (%)	90.56 ± 0.05 c	99.96 ± 0.06 a	95.62 ± 0.13 b	98.99 ± 0.10 a	98.82 ± 0.04 a
Protein (%)	0.30 ± 0.00 ^b^	0.18± 0.00 ^c^	0.13 ± 0.01 ^c^	0.61 ± 0.01 ^a^	0.11 ± 0.27 ^c^
Degree of methylesterification	27.35 ± 0.51 ^b^	38.94 ± 3.10 ^a^	32.40 ± 0.91 ^ab^	28.21 ± 0.64 ^b^	27.73 ± 0.49 ^b^
Monosaccharide composition molar ratio (%)					
Fuc	3.74 ± 0.02 ^a^	1.12 ± 0.05 d	1.36 ± 0.06 c	1.63 ± 0.02 b	1.48 ± 0.05 c
Ara	18.28 ± 0.24 c	25.47 ± 0.27 a	24.96 ± 0.21 b	24.31 ± 0.05 b	25.60 ± 0.06 a
Rha	3.84 ± 0.08 c	6.20 ± 0.08 a	6.07 ± 0.07 a	5.78 ± 0.01 b	6.28 ± 0.05 a
Gal	24.62 ± 0.36 c	26.15 ± 0.19 b	26.95 ± 0.09 a	25.83 ± 0.03 b	27.12 ± 0.07 a
Glc	24.93 ± 0.28 a	12.77 ± 0.11 d	13.43 ± 0.03 c	14.76 ± 0.04 b	12.87 ± 0.06 d
Xyl	7.62 ± 0.18 a	1.29 ± 0.04 c	1.54 ± 0.05 c	1.92 ± 0.05 b	1.76 ± 0.05 d
Man	2.64 ± 0.06 a	1.04 ± 0.07 b	1.20 ± 0.07 b	1.18 ± 0.04 b	1.22 ± 0.05 b
GalA	13.27 ± 0.22 d	24.28 ± 0.08 a	22.82 ± 0.07 b	23.03 ± 0.03 b	22.05 ± 0.11 c
GlcA	1.07 ± 0.02 b	1.67 ± 0.06 a	1.67 ± 0.04 a	1.57 ± 0.02 a	1.63 ± 0.06 a
Molecular weight distribution					
Mw (kDa)	1079.9 ± 0.65 a	824.4 ± 0.05 b	757.0 ±0.09 c	690.7 ± 0.05 e	698.3 ± 0.05 ^d^
Mn (kDa)	352.1 ± 0.08 a	157.0 ± 0.05 b	133.7 ± 0.05 c	125.1 ± 0.09 e	126.5 ± 0.09 ^d^
Polydispersity (Mw/Mn)	3.06 ± 0.00 d	5.25 ± 0.00 c	5.66 ± 0.00 a	5.52 ± 0.00 b	5.52 ± 0.00 ^b^

Values are presented as mean ± SD. Different superscript letters (a, b, c, d, e) in the same row indicate significant differences (*p* < 0.05). Fucose (Fuc), Arabinose (Ara), Rhamnose (Rha), Galactose (Gal), Glucose (Glc), Xylose (Xyl), Mannose (Man), Galacturonic acid (GalA), and Glucuronic acid (GlcA).

**Table 2:** Chemical shifts of ^1^H and ^13^C for each sugar residue of uFCTP.

Code	Glycosyl Residues	Chemical Shifts (ppm)					
		H1/C1	H2/C2	H3/C3	H4/C4	H5/C5	H6/C6
A	→4)-α-D-GalpA-6-OMe-(1→	4.85	3.6	3.89	4.35	4.98	/
		98.53	67.72	68.39	79.01	70.5	170.63
B	→4)-α-D-GalpA-(1→	4.89	3.73	3.85	4.33	n.d	/
		100.35	68.56	68.41	79.05	n.d	175.64
C	→4)-β-D-Galp-(1→	4.53	3.57	3.66	4.06	3.61	3.71
		104.29	72	73.68	77.6	74.45	60.68
D	α-L-Araf-(1→	5.13	4.1	3.9	4.02	3.61	/
		109.18	81.27	76.67	83.78	61.21	/
E	→4)-α-D-Glcp-(1→	5.29	3.53	3.85	3.55	3.66	3.68
		99.73	71.75	73.24	77.2	72.07	60.5
F	→3,6)-β-D-Galp-(1→	4.42	3.29	3.64	4.04	3.89	3.82, 3.94
		103.08	72.69	80.03	68.47	73.34	69.39
G	→5)-α-L-Araf-(1→	4.98	4.02	3.84	3.94	3.69, 3.78	/
		107.36	80.75	76.5	83.92	66.79	/
H	→3,5)-α-L-Araf-(1→	5.07	4.26	3.86	3.99	3.72, 3.84	/
		107.44	79.6	83.83	83.94	66.43	/
I	β-D-Glcp-(1→	4.55	3.17	3.64	3.44	3.53	3.66
		95.67	73.36	72.53	71.87	74.24	60.94

Note: /, not applicable; n.d., not detected.

## Data Availability

The original contributions presented in the study are included in the article/Supplementary Material, further inquiries can be directed to the corresponding authors.

## Supplementary Materials

The following supporting information can be downloaded at: https://www.mdpi.com/article/10.3390/foods15152749/s1, Figure S1: Effects of various factors on the extraction yield of FCTP by hot-water extraction (HWE); Figure S2: 3D plot of the effects of different factors on the yield of FCTP obtained by hot-water extraction (HWE); Figure S3: 3D plot of the effects of different factors on the yield of FCTP obtained by enzyme-assisted water extraction (EAE); Figure S4: The comparative growth data for FCTP-120W20, FCTP-120W45, FCTP-240W20 and FCTP-240W45. Data are presented as mean ± SD (*n* = 3). The differences were analyzed using one-way ANOVA followed by Duncan’s multiple-range test. Different letters indicate significant differences (*p* < 0.05); Figure S5: Top 30 positive correlation of proteome and metabolome for uFCTP group vs. CON group. uFCTP: ultrasonically degraded; CON: no carbon (blank control); Figure S6: Top 30 positive correlation of proteome and metabolome for nFCTP vs. CON group. nFCTP: native FCTP; CON: no carbon (blank control). Table S1: Box-Behnken optimization of FCTP extracted using hot-water extraction (HWE); Table S2: Box-Behnken optimization of FCTP extracted using enzyme-assisted water extraction (EAE); Table S3: The glycosidic linkage pattern of uFCTP.
