# Supplementary material for: Ultrasonic Degradation Improves the In Vitro Utilization of Oolong Tea Pectic Polysaccharides: Structure Characterization and Multi-Omics Insights
Source: Foods. 2026 Aug 5;15(15):2749. doi: 10.3390/foods15152749 (PMC13465410; doi:10.3390/foods15152749)
Supplement: Supplementary file 1 [file foods-15-02749-s001.zip › foods-4431301-supplementary.pdf]

Supplementary materials

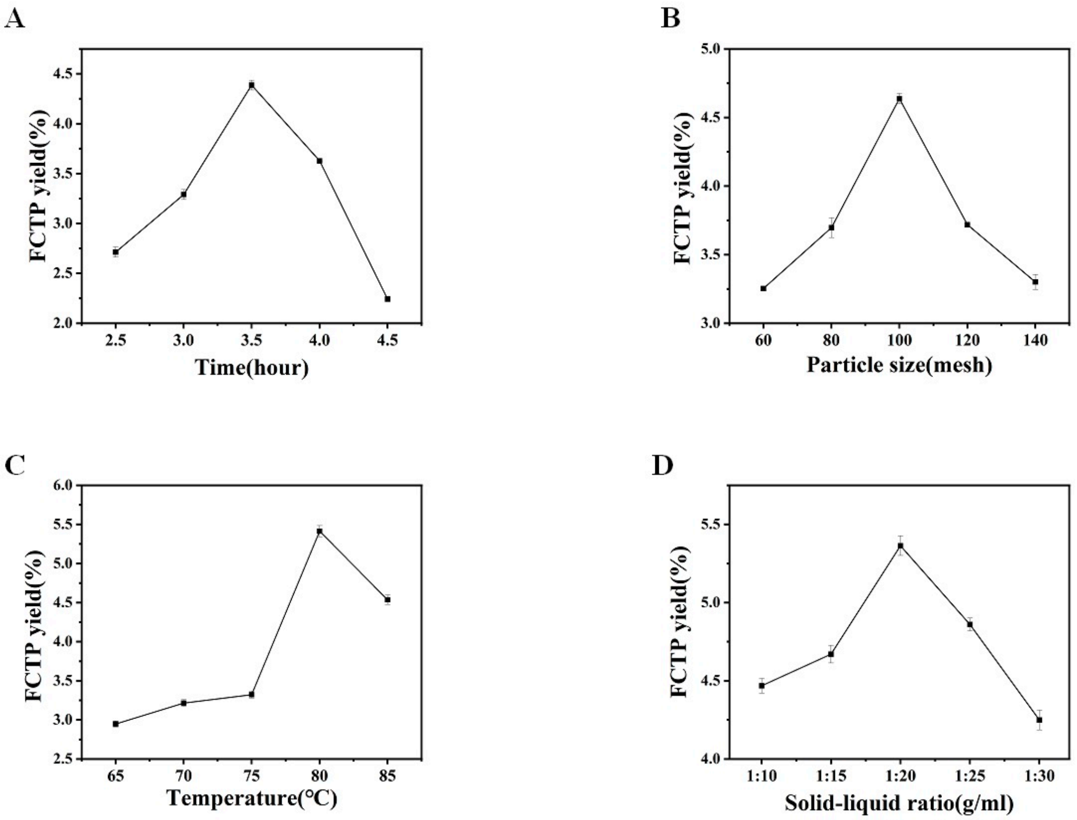

Figure S1. Effects of various factors on the extraction yield of FCTP by hot-water extraction (HWE).

Table S1. Box–Behnken optimization of FCTP extracted using hot-water extraction (HWE).

No.	A<br>Time (min)	B<br>Particle Size(mesh)	C<br>Temperature (℃)	D<br>Solid–liquid ratio (g/mL)	FCTP yield (%)
1	210	120	75	25	7.39±0.02
2	210	100	80	25	4.76±0.01
3	210	120	85	25	8.52±0.05
4	240	100	80	20	5.28±0.01
5	240	120	75	20	4.96±0.01
6	210	100	80	15	5.21±0.02
7	180	120	85	20	4.86±0.04
8	210	120	80	20	8.74±0.01
9	210	120	80	20	8.74±0.01
10	180	120	80	25	6.44±0.02
11	210	120	80	20	8.74±0.02
12	210	100	85	20	4.71±0.03
13	210	120	85	15	6.03±0.02
14	180	120	75	20	6.22±0.04
15	240	120	80	15	5.41±0.05
16	210	120	80	20	8.79±0.03
17	180	100	80	20	4.20±0.06
18	180	120	80	15	6.61±0.06
19	210	120	80	20	8.74±0.08
20	180	140	80	20	5.87±0.06
21	240	140	80	20	5.60±0.06
22	210	140	85	20	6.32±0.07
23	210	140	75	20	5.39±0.07
24	240	120	85	20	5.35±0.05
25	210	140	80	15	4.90±0.07
26	210	140	80	25	8.81±0.05
27	240	120	80	25	4.66±0.06
28	210	120	75	15	6.05±0.06
29	210	100	75	20	4.22±0.10

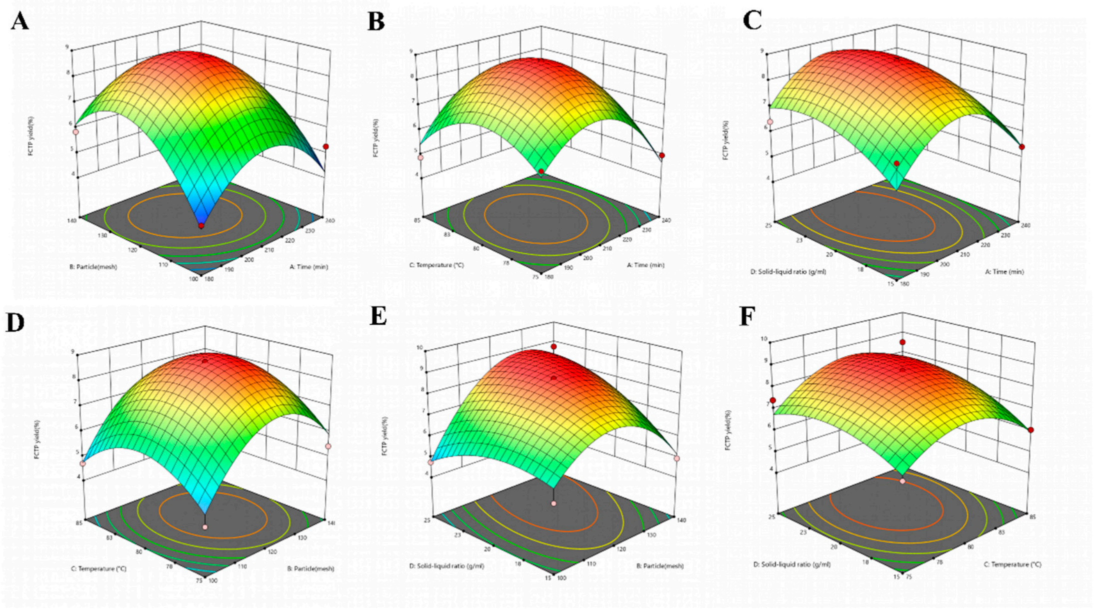

Figure S2. 3D plot of the effects of different factors on the yield of FCTP obtained by hot-water extraction (HWE).

Table S2. Box–Behnken optimization of FCTP extracted using enzyme-assisted water extraction (EAE).

No.	A<br>Solid–liquid ratio (g/mL)	B<br>Time (min)	C<br>Temperature (°C)	D<br>Enzyme dosage (%)	FCTP yield (%)
1	25	100	55	0.4	10.42±0.04
2	30	60	55	0.6	10.99±0.01
3	25	80	60	0.4	9.03±0.02
4	25	80	55	0.6	11.14±0.03
5	25	60	55	0.8	10.43±0.02
6	20	80	55	0.8	10.89±0.02
7	20	100	55	0.6	9.93±0.03
8	30	80	55	0.4	10.86±0.00
9	25	60	50	0.6	8.75±0.00
10	20	80	60	0.6	10.37±0.05
11	25	80	55	0.6	12.43±0.01
12	30	80	50	0.6	10.83±0.04
13	25	80	55	0.6	12.60±0.03
14	30	80	55	0.8	10.97±0.03
15	30	80	60	0.6	10.17±0.01
16	25	60	55	0.4	10.11±0.03
17	25	100	50	0.6	9.75±0.05
18	25	100	60	0.6	10.11±0.02
19	20	80	55	0.4	9.13±0.01
20	25	80	60	0.8	8.16±0.02
21	30	100	55	0.6	11.37±0.01
22	25	80	50	0.4	9.89±0.02
23	20	60	55	0.6	12.85±0.05
24	25	100	55	0.8	10.95±0.06
25	20	80	50	0.6	9.13±0.02
26	25	60	60	0.6	9.07±0.04
27	25	80	50	0.8	9.20±0.03

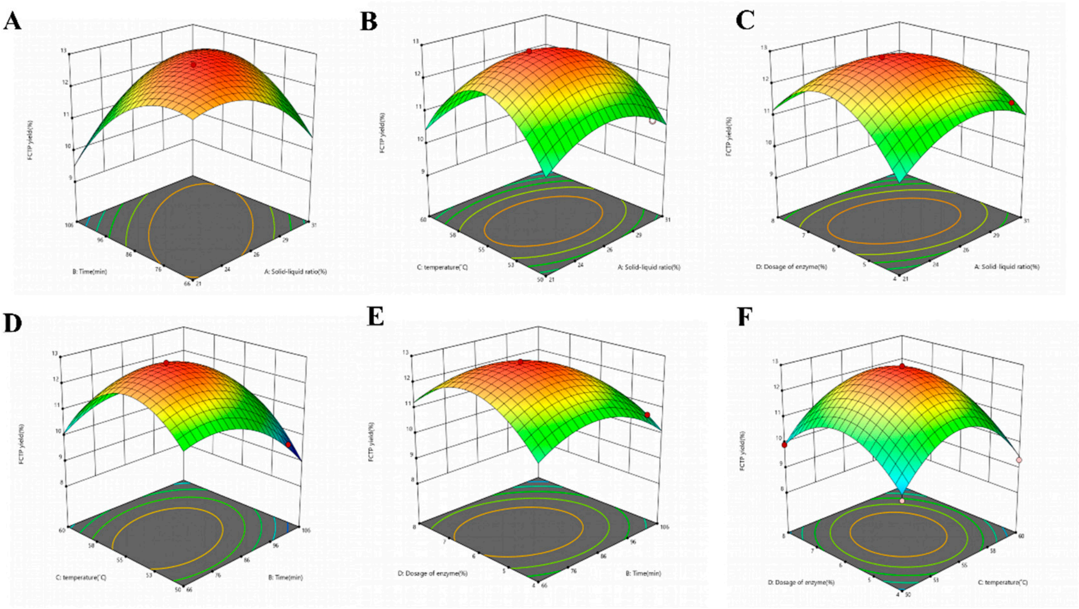

Figure S3. 3D plot of the effects of different factors on the yield of FCTP obtained by enzyme-assisted water extraction (EAE).

Table S3. The glycosidic linkage pattern of uFCTP.

RT	Methylated sugar	Mass fragments （m/z）	Molar ratio(%)	Type of linkage
10.869	1,4-di-O-acetyl-2,3,5-tri-O-methyl arabinitol	71,87,102,118,129,145,161	8.56	t-Ara(f)
11.403	1,5-di-O-acetyl-6-deoxy-2,3,4-tri-O-methyl mannitol	59,72,89,102,115,118,131,145,162,175	1.98	t-Rha(p)
13.812	1,3,4-tri-O-acetyl-2,5-di-O-methyl arabinitol	87,99,113,118,129,201,233	1.83	3-Ara(f)
13.928	1,5-di-O-acetyl-2,3,4,6-tetra-O-methyl glucitol	87,102,118,129,145,161,162,205	1.94	t-Glc(p)
14.455	1,5-di-O-acetyl-2,3,4,6-tetra-O-methyl galactitol	73,89,102,118,147,162,163,207	2.81	t-Gal(p)-UA
14.455	1,5-di-O-acetyl-2,3,4,6-tetra-O-methyl galactitol	87,102,118,129,145,161,162,205	1.73	t-Gal(p)
14.831	1,4,5-tri-O-acetyl-2,3-di-O-methyl arabinitol	87,102,118,129,162,189	3.52	5-Ara(f)
16.412	1,3,5-tri-O-acetyl-2,4,6-tri-O-methyl glucitol	87,101,118,129,161,202,234	2.39	3-Glc(p)
16.898	1,3,4,5-tetra-O-acetyl-2-O-methyl arabinitol	85,99,118,127,159,201,261	1.81	3,5-Ara(f)
16.946	1,2,4,5-tetra-O-acetyl-6-deoxy-3-O-methyl mannitol	88,101,117,130,143,190,203	1.95	2,4-Rha(p)
17.131	1,4,5-tri-O-acetyl-2,3,6-tri-O-methyl galactitol	87,99,102,115,118,131,162,175, 235	28.90	4-Gal(p)-UA
17.131	1,4,5-tri-O-acetyl-2,3,6-tri-O-methyl galactitol	87,102,113,118,129,162,233	26.83	4-Gal(p)
17.267	1,4,5-tri-O-acetyl-2,3,6-tri-O-methyl glucitol	87,102,113,118,129,162,233	5.71	4-Glc(p)
18.547	1,5,6-tri-O-acetyl-2,3,4-tri-O-methyl glucitol	87,99,102,118,129,162,189,233	1.80	6-Glc(p)
18.643	1,3,4,5-tetra-O-acetyl-2,6-di-O-methyl galactitol	87,118,129,143,185,203,305	1.48	3,4-Glc(p)-UA
21.113	1,4,5,6-tetra-O-acetyl-2,3-di-O-methyl glucitol	85,102,118,127,159,162,201,261	1.50	4,6-Glc(p)
21.606	1,3,5,6-tetra-O-acetyl-2,4-di-O-methyl galactitol	87,101,118,129,160,189,234	5.27	3,6-Gal(p)

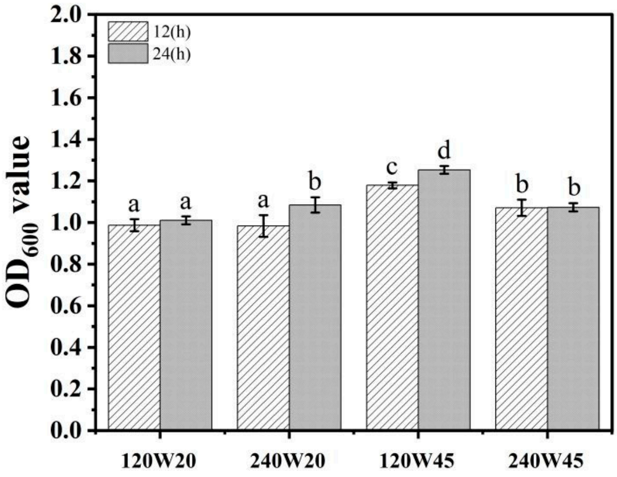

Figure S4. The comparative growth data for FCTP-120W20, FCTP-120W45, FCTP-240W20 and FCTP-240W45. Data are presented as mean ± SD (n = 3). The differences were analyzed using one-way ANOVA followed by Duncan's multiple-range test. Different letters indicate significant differences ($p < 0.05$).

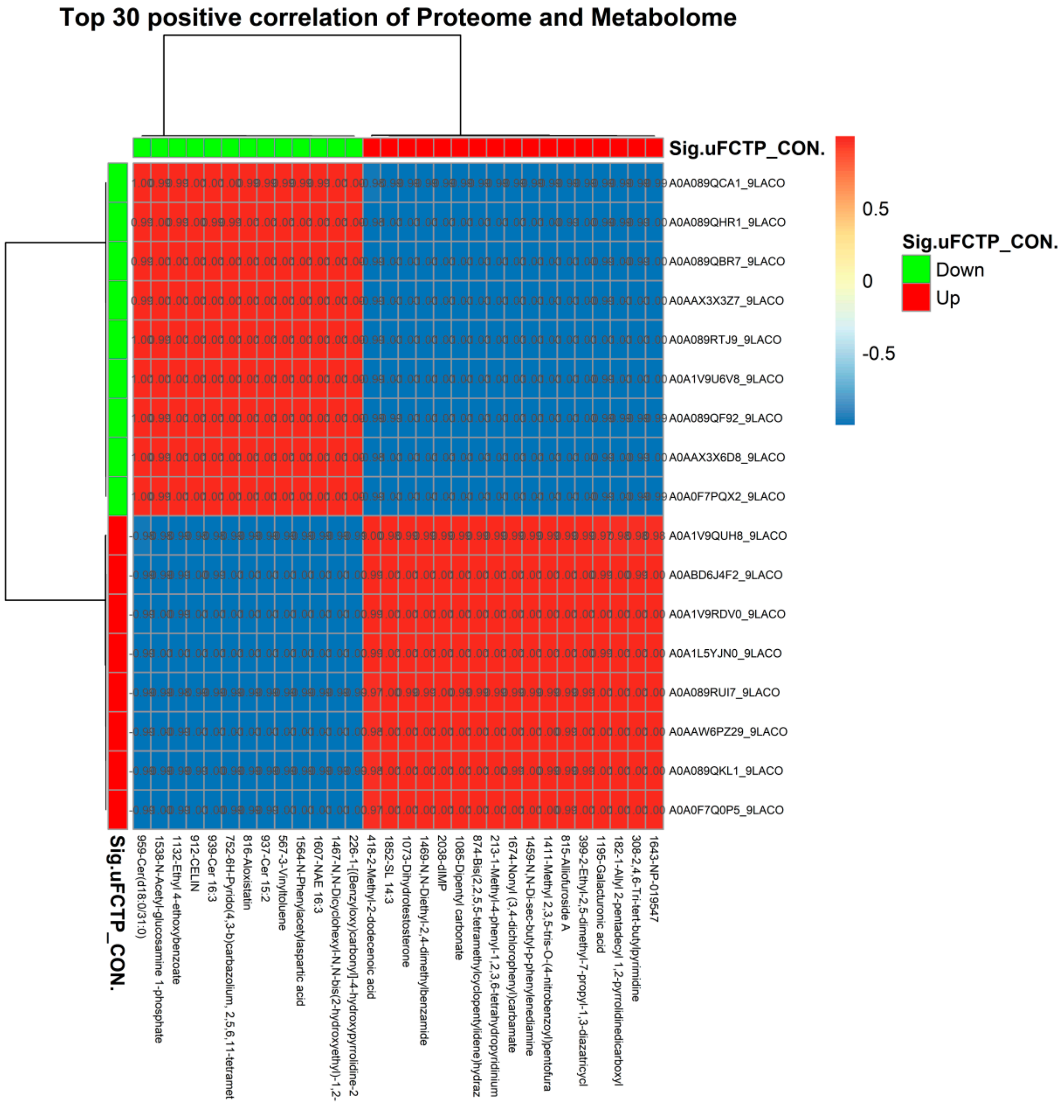

Figure S5. Top 30 positive correlation of proteome and metabolome for uFCTP group vs. CON group. uFCTP: ultrasonically degraded; CON: no carbon (blank control).

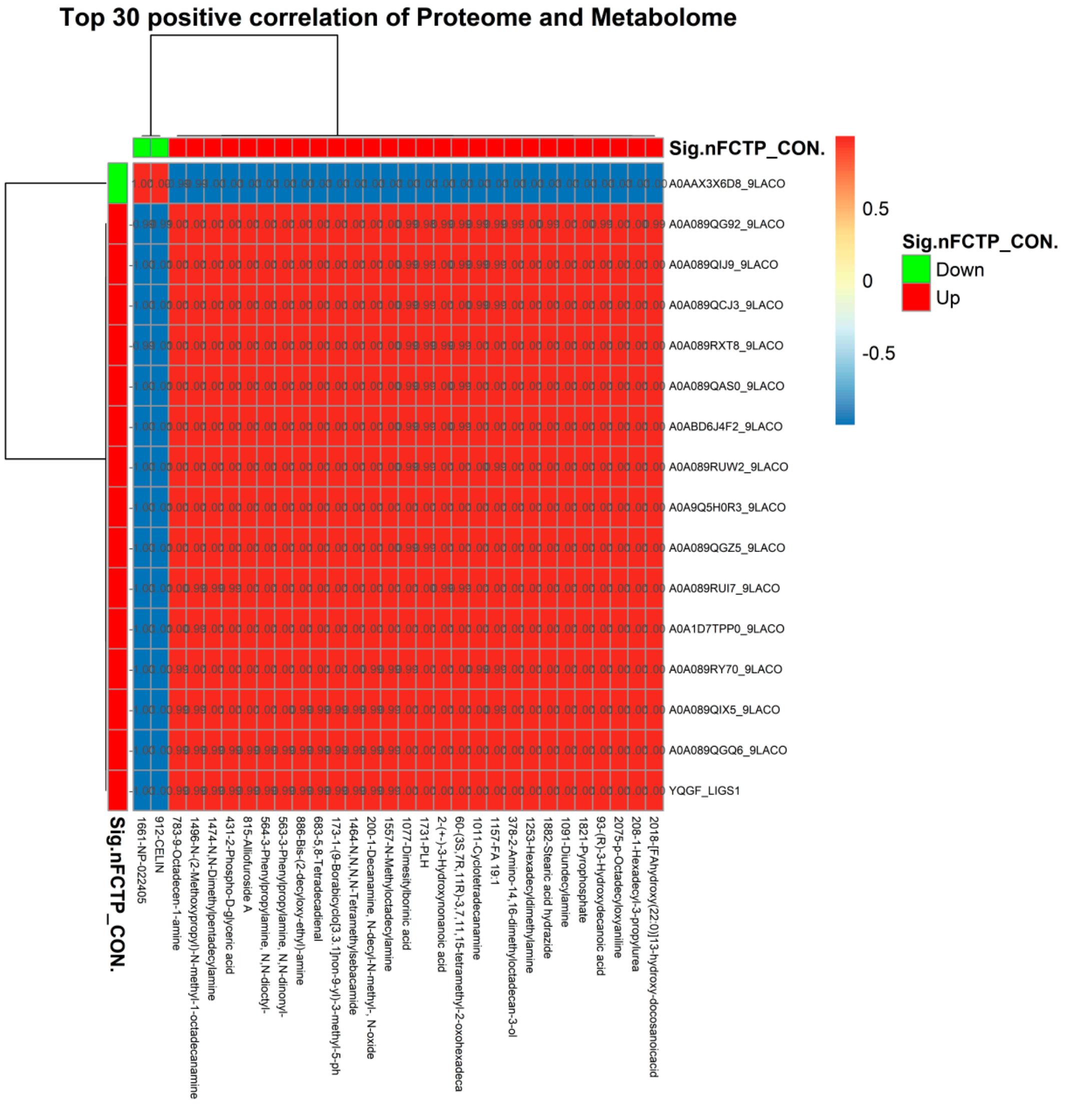

Figure S6. Top 30 positive correlation of proteome and metabolome for nFCTP vs. CON group.

nFCTP: native FCTP; CON: no carbon (blank control).
